# Клинические и лабораторные характеристики резистентности к антидиуретическому гормону, обусловленной новой гомозиготной мутацией p.R113C в гене AQP2

**DOI:** 10.14341/probl13188

**Published:** 2023-05-12

**Authors:** Н. А. Макрецкая, У. С. Нанзанова, И. Р. Хамаганова, Е. Р. Еремина, А. Н. Тюльпаков

**Affiliations:** Медико-генетический научный центр имени академика Н.П. Бочкова; Детская республиканская клиническая больница; Детская республиканская клиническая больница; Республиканский перинатальный центр МЗ РБ; Бурятский государственный университет имени Доржи Банзарова; Научный центр проблем здоровья семьи и репродукции человека; Медико-генетический научный центр имени академика Н.П. Бочкова

**Keywords:** резистентность к АДГ, AQP2, аквапорин-2, буряты, эффект основателя

## Abstract

Врожденный нефрогенный несахарный диабет (ННД, резистентность к антидиуретическому гормону (АДГ)) — редкое наследственное заболевание, характеризующееся нечувствительностью дистальных отделов нефрона к антидиуретическому эффекту вазопрессина. Основными клиническими проявлениями заболевания являются выраженная полиурия с гипостенурией и никтурией и полидипсия. В большинстве случаев, около 90%, ННД представляет собой Х-сцепленное рецессивное заболевание, вызванное мутациями в гене рецептора вазопрессина (AVPR2). Остальные 10% случаев опосредованы инактивирующими мутациями в гене аквапорина-2 (AQP2) и имеют аутосомно-рецессивный или аутосомно-доминантный типы наследования. Зарегистрированные на сегодняшний день нуклеотидные изменения в гене AQP2 носят спорадический характер, отсутствуют данные о наличии «частых» мутаций и распространенности заболевания как среди мировой популяции, так и среди отдельных этнических групп. В данной работе впервые в мировой литературе представлено описание 12 случаев резистентности к АДГ, обусловленной новой гомозиготной мутацией p.R113C в гене AQP2, среди коренного населения Республики Бурятия.

## Abstract (en)

Congenital nephrogenic diabetes insipidus (CNDI, arginine vasopressin resistance) is a rare inherited disorder characterized by insensitivity of the kidney to the antidiuretic effect of vasopressin. NDI is clinically characterized by polyuria with hyposthenuria and nocturia and polydipsia. In the majority of cases, about 90%, nephrogenic diabetes insipidus is an X-linked recessive disorder caused by mutations in the AVP V2 receptor gene (AVPR2). In the remaining cases, about 10%, the disease is autosomal recessive or dominant and, for these patients, mutations in the aquaporin 2 gene (AQP2) have been reported. To date, the nucleotide variants registered in AQP2 were sporadic, there is no data on the presence of «frequent» mutations and the prevalence of the disease both among the global population and among individual ethnic groups. In this paper, we describe 12 cases of arginine vasopressin resistance caused by a new homozygous mutation p.R113C in AQP2 presented among the indigenous population of the Republic of Buryatia.

## АКТУАЛЬНОСТЬ

Международным коллективом автором в 2022 г. предложена новая концепция названий для несахарного диабета, основанная на этиопатогенезе: для центрального несахарного диабета — «дефицит вазопрессина (антидиуретического гормона (АДГ))», для нефрогенного несахарного диабета (ННД) — «резистентность к вазопрессину (АДГ)» [1]. Обновленная номенклатура призвана повысить уровень понимания патологии врачами-неэндокринологами и снизить риски необоснованного назначения десмопрессина.

Резистентность к АДГ (ННД) представляет собой гетерогенную группу заболеваний, характеризующихся нарушением резорбции воды собирательными трубочками нефронов почек. На сегодняшний день описано два наследственных варианта развития врожденного ННД: обусловленный инактивирующими мутациями в гене AVPR2 (OMIM #300538), которые приводят к нарушению чувствительности рецептора к действию вазопрессина (AVP), и в гене AQP2 (OMIM #107777), участвующем в процессе реабсорбции воды в просветах собирательных трубочек [2][3].

По данным литературы, около 90% всех наследственных форм резистентности к АДГ вызвано мутациями в гене AVPR2 с Х-сцепленным рецессивным типом наследования [4]. Остальные 10% случаев обусловлены патологическими изменениями в гене AQP2 с аутосомно-рецессивным или аутосомно-доминантным типами наследования заболевания [2][5]. К настоящему моменту описано более 70 патогенных вариантов, расположенных по всей протяженности гена AQP2, в 74 семьях (http://www.hgmd.cf.ac.uk), что свидетельствует о единичном распространении отдельных мутаций.

Известно, что в относительно изолированных этнических группах накопление отдельных мутантных аллелей приводит к распространению этноспецифических заболеваний.

В данном исследовании нами представлено описание 12 пациентов — этнических бурятов с резистентностью к АДГ, обусловленной гомозиготной мутацией p.R113C в гене AQP2.

## ОПИСАНИЕ СЛУЧАЕВ

В исследование включены 12 пациентов из 11 семей с диагнозом «нефрогенный несахарный диабет» (5 девочек, 7 мальчиков). Этническая принадлежность обследуемых — буряты. Все пациенты обследованы в Республиканской детской клинической больнице, г. Улан-Удэ.

Первоначально ННД был заподозрен у девочки 4 лет (пациент №1, табл. 1). Учитывая пол ребенка и отсутствие клинических проявлений заболевания у кого-либо из родителей, было предположено наличие рецессивного варианта ННД, и при секвенировании гена AQP2 обнаружена гомозиготная мутация c.337C>T p.R113C (рис. 1). В период с 2014 по 2020 гг. схожая клиническая симптоматика была отмечена еще у 11 пациентов той же этнической группы, которым уже прицельно проводилось исследование гена AQP2, выявлена аналогичная мутация. Таким образом, по результатам молекулярно-генетического исследования гомозиготный вариант c.337C>T p.R113C в гене AQP2 обнаружен у 12 пробандов из 11 семей (5 девочек, 7 мальчиков) (табл. 1).

Геномную ДНК выделяли из лейкоцитов периферический крови наборами PureLink® Genomic DNA Mini Kit (Thermo Scientific, Waltham, MA, USA). Применялся метод секвенирования по Сэнгеру на секвенаторе Genetic Analyzer Model 3130 (Thermo Scientific, Waltham, MA, USA) с использованием следующего набора праймеров:

AQ2_1F: GCCTTGAGAAAGAGAGCGATAG;

AQ2_1R: CAGAGCCCATCCCTCCCATCTC;

AQ2_2F: CGTCTGGCAAGCCCAGGTGTTC;

AQ2_3F: CCTTTAGGCTGAGGTCAAG;

AQ2_4R: CACGTCCAGGAAGCAGCTACTC.

Оценка патогенности варианта нуклеотидной последовательности проводилась согласно международным и российским рекомендациям [6][7]. Нумерация кодирующей последовательности гена AQP2 дана по референсу NM_000486.3 (http://www.ncbi.nlm.nih.gov/genbank). Для сравнения частоты нуклеотидного варианта использованы данные gnomAD (https://gnomad.broadinstitute.org/) [8].

Ведущими клиническими симптомами у всех обследуемых пациентов с гомозиготной мутацией p.R113C в гене AQP2 являлись полиурия и полидипсия до нескольких литров сутки (табл. 1). При сборе анамнестических данных установлено, что описанные жалобы беспокоили с раннего возраста (Ме возраста начала клинических проявлений составила 5 [ 3; 9] мес), со слов родителей, среднее количество выпиваемой жидкости к возрасту 1 года составляло 2–3 л/сут, в общем анализе мочи амбулаторно выявлено снижение относительной плотности мочи до 1000–1003 г/л. Отмечалось постепенное нарастание симптоматики с возрастом ребенка. У 3 пациентов (№ 3, 7, 10) также на первом году жизни наблюдались эпизоды субфебрилитета до 37,6°С, без признаков присоединения интеркуррентных заболеваний (за дополнительным обследованием не обращались), купировавшиеся самостоятельно на фоне дотации жидкости. Медикаментозная терапия обследуемым пациентам не назначалась.

Ме возраста постановки диагноза составила 3 [ 2,25; 4,5] года. На момент госпитализации у всех пациентов отмечалась полиурия со снижением относительной плотности мочи (Ме 1001 [ 1001; 1002] г/л). В биохимическом анализе крови в 3 случаях выявлено повышение уровня натрия (№ 5, 7, 8), Ме натрия составила 144 [ 139; 146] ммоль/л. Уровни калия (Ме 4,3 [ 4,1; 4,4] ммоль/л), глюкозы (Ме 4,3 [ 4,1; 4,7] ммоль/л) и кальция (Ме 2,5 [ 2,4; 2,6] ммоль/л) соответствовали референсным интервалам. У 7 пациентов выявлено повышение уровня осмолярности крови, Ме 301,5 [ 293; 307] мОсм/кг. По техническим причинам исследование уровня осмолярности мочи выполнено не было. По данным ультразвукового исследования почек в 6 случаях диагностирована пиелоэктазия. Диагноз «нефрогенный несахарный диабет» устанавливался на основании проведения пробы с десмопрессином (за исключением пациента №6–2): на фоне приема препарата не выявлено значимого повышения уровня относительной плотности мочи (Ме 1002 [1001–1003]). Пациенту 6–2 диагноз установлен на основании клинической картины и отягощенной наследственности по данному заболеванию.

**Table:** Таблица 1. Клинические и лабораторные данные пациентов

№	Пол	Возраст манифестации, месяцы	Возраст постановки диагноза, годы	Полиурия/ полидипсия	Диурез, мл	Относительная плотность мочи, г/л	Na сыворотки, ммоль/л	К сыворотки, ммоль/л	Глюкоза, ммоль/л	Са общий, ммоль/л	Осмолярность крови мОсм/кг	Пиелоэктазия	Субфебрилитет	Отягощенная наследственность
1	Жен.	4	4	да	5320	1002	144	4,1	4,0	2,4	302	да	нет	нет
2	Жен.	2	3	да	4460	1001	139	4,3	4,6	2,5	294	нет	нет	нет
3	Муж.	10	2	да	3570	1003	143	4,2	4,1	2,3	301	нет	да	нет
4	Муж.	12	3	да	4505	1000	137	4,5	4,2	2,7	290	нет	нет	нет
5	Муж.	7	1,5	да	3780	1001	146	4,3	4,8	2,5	307	да	нет	нет
6–1	Жен.	3	5	да	5640	1002	145	4,0	4,1	2,3	304	да	нет	да, сибс 6–2
6–2	Муж.	2	3	да	3830	1001	138	4,4	4,3	2,7	291	нет	нет	да, сибс 6–1
7	Муж.	3	2	да	3540	1001	153	4,5	5,0	2,2	322	да	да	нет
8	Жен.	11	7	да	4580	1001	147	3,8	4,1	2,6	309	да	нет	нет
9	Муж.	6	4	да	4260	1003	143	3,9	4,2	2,5	300	нет	нет	нет
10	Муж.	8	5	да	4130	1002	138	4,3	4,4	2,4	291	да	да	нет
11	Жен.	3	3	да	3890	1002	145	4,4	4,7	2,6	306	нет	нет	нет

**Figure:**
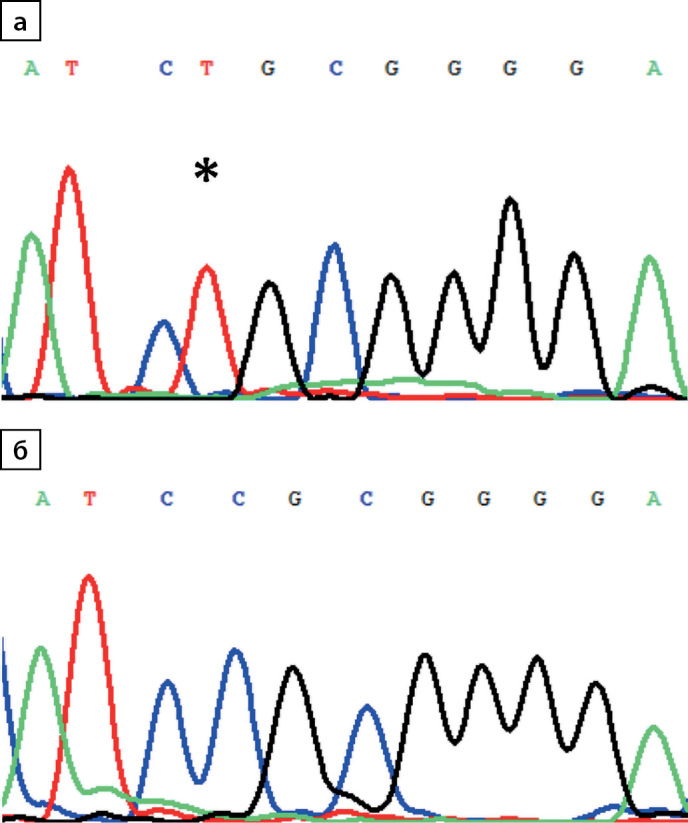
Рисунок 1. Электрофореграмма фрагмента последовательности экзона 1 гена AQP2: а) гомозиготная транзиция c.337C>T (*) с заменой кодона аргинина (CGC) на цистеин (TGC) в положении 113 (p.R113C); б) последовательность дикого типа.

## ОБСУЖДЕНИЕ

Ген AQP2 (OMIM #107777) картирован на длинном плече хромосомы 12 (12q13.12) в 1993 г. (Sasaki S. и соавт.), состоит из четырех экзонов и трех интронов и кодирует белок аквапорин-2 (AQP2) [9][10].

AQP2 состоит из 271 аминокислоты с молекулярной массой 28 837 Да, в своем составе имеет N- и С-концевые домены, три экстрацеллюлярные петли, две интрацеллюлярные петли и шесть трансмембранных доменов. AQP2 регулирует реабсорбцию воды в ответ на стимуляцию AVP. При уменьшении объема плазмы или повышении ее осмоляльности в нейрогипофизе секретируется AVP и, связываясь с рецептором AVPR2 в собирательных трубочках почек, увеличивает внутриклеточную концентрацию циклического аденозинмонофосфата. Данный процесс стимулирует транспортировку AQP2 к апикальной плазматической мембране и ингибирует его интернализацию, опосредованную эндоцитозом. Как результат, происходит накопление AQP2 в апикальной плазматической мембране, что позволяет воде двигаться по осмотическому градиенту через мембрану в интерстиций, а затем в кровоток [11].

Впервые клиническая картина инактивирующих мутаций в гене AQP2 описана Р. Deen и соавт. в 1994 г. [12]. Ведущими клиническими проявлениями заболевания являются выраженная полиурия с гипостенурией и никтурией и полидипсия до 10–20 л/сут, развивающиеся на первом году жизни [5]. Кроме того, у ряда пациентов отмечается развитие неспецифических симптомов заболевания, таких как субфебрилитет, раздражительность, снижение аппетита, рвота и вялость, что может приводить к задержке роста и снижению массы тела у детей [6]. При лабораторном обследовании характерно снижение относительной плотности мочи, также возможно повышение уровня натрия и осмолярности плазмы на фоне ограничения жидкости [5][11]. У пациентов старшего возраста возможно развитие таких осложнений, как ортостатическая гипотензия, гидронефроз, мегауретер [11].

На сегодняшний день не разработано патофизиологического лечения резистентности к АДГ. Рекомендации по ведению таких пациентов направлены на восполнение потери жидкости с мочой адекватным количеством выпитой, в сочетании с низкосолевой диетой. Кроме того, возможно применение нестероидных противовоспалительных препаратов (НПВС) и диуретиков [13, 14]. НПВС, такие как ибупрофен и индометацин, улучшают способность концентрировать мочу и уменьшают ее объем на 25–50%, а комбинация с гидрохлоротиазидом оказывает аддитивный эффект [13][14]. Тиазидные диуретики эффективно снижают диурез при соблюдении диеты с очень низким содержанием натрия [13]. Калийсберегающие диуретики, такие как амилорид, могут иметь аддитивный эффект с тиазидными диуретиками через механизм ингибирования потери калия, вызванной тиазидами [14]. Однако для всех перечисленных вариантов терапии описан эффект ускользания [13][14].

Ранее в России описан клинический случай ННД, обусловленный мутацией p. D150E в гене AQP2 [15], патогенность которой была затем доказана функциональными исследованиями in vitro [16]. Выявленный в ходе настоящего исследования нуклеотидный вариант hg38_chr12:49951167C>T (c.337C>T p.R113C) приводит к замене аргинина на цистеин в положении 113. Ранее данная мутация у пациентов с резистентность к АДГ не описана. По данным базы gnomAD, общая частота для данного варианта составляет 0,00001243 (3:241 284), все 3 случая выявлены в гетерозиготном состоянии: 1 — южно-азиатская группа, 1 — восточно-азиатская группа, 1 — латиноамериканская группа [17]. Согласно критериям ACMG, обнаруженный вариант нуклеотидной последовательности может быть расценен как патогенный с уровнем значимости РМ2, РM1, PP3, PP4 [6][7]. Аргинин в 113 положении является частью консервативного интегринсвязывающего RGD-домена на второй экстрацеллюлярной петле. В исследованиях in vitro показано, что изменения в данном регионе могут приводить к нарушению процесса транспорта AQP2 к апикальной мембране клетки [18].

На сегодняшний день в мировой литературе отсутствуют данные о распространенности резистентности к АДГ, обусловленной мутациями в гене AQP2. Описанные ранее случаи данного заболевания являлись спорадическими, также не было выявлено каких-либо часто встречающихся мутаций для различных популяций. В нашем исследовании нуклеотидный вариант hg38_chr12:49951167C>T (c.337C>T p.R113C) идентифицирован в 12 случаях среди коренного населения Бурятии, что позволяет отнести данное изменение к основным причинам развития резистентности к АДГ в данной этнической группе и предположить наличие эффектов «основателя» и «бутылочного горлышка» в распространении варианта p.R113C среди коренного населения Республики Бурятия.

## ЗАКЛЮЧЕНИЕ

Впервые для мировой литературы описана клиническая характеристика резистентности к АДГ, обусловленная мутацией p.R113C в гене AQP2. Высокая распространенность данного заболевания среди коренного населения Бурятии позволит в дальнейшем проводить медико-генетическое консультирование семей в регионе, повысить настороженность врачей в отношении резистентности к АДГ, улучшит раннюю диагностику этого состояния, в том числе с использованием методов пренатальной диагностики.

## ДОПОЛНИТЕЛЬНАЯ ИНФОРМАЦИЯ

Источники финансирования. Данная работа поддержана советом по грантам Президента Российской Федерации, номер гранта МК-5272.2022.3.

Конфликт интересов. Авторы декларируют отсутствие явных и потенциальных конфликтов интересов, связанных с содержанием настоящей статьи.

Участие авторов. Макрецкая Н.А. — существенный вклад в дизайн исследования, сбор материала, анализ полученных данных, написание текста; Нанзанова У.С. — сбор материала, анализ полученных данных; Хамаганова И.Р. — сбор материала, анализ полученных данных; Еремина Е.Р. — сбор материала, анализ полученных данных; Тюльпаков А.Н. — концепция и дизайн исследования, внесение в рукопись существенной правки с целью повышения научной ценности статьи. Все авторы одобрили финальную версию статьи перед публикацией, выразили согласие нести ответственность за все аспекты работы, подразумевающую надлежащее изучение и решение вопросов, связанных с точностью или добросовестностью любой части работы.

Согласие пациента. Добровольные информированные согласия пациентов и их законных представителей на публикацию в журнале «Проблемы эндокринологии» получены.
